# Detection of Impaired Cerebral Autoregulation Using Selected Correlation Analysis: A Validation Study

**DOI:** 10.1155/2017/8454527

**Published:** 2017-01-31

**Authors:** Martin A. Proescholdt, Rupert Faltermeier, Sylvia Bele, Alexander Brawanski

**Affiliations:** Department of Neurosurgery, University Hospital Regensburg, Regensburg, Germany

## Abstract

Multimodal brain monitoring has been utilized to optimize treatment of patients with critical neurological diseases. However, the amount of data requires an integrative tool set to unmask pathological events in a timely fashion. Recently we have introduced a mathematical model allowing the simulation of pathophysiological conditions such as reduced intracranial compliance and impaired autoregulation. Utilizing a mathematical tool set called selected correlation analysis (sca), correlation patterns, which indicate impaired autoregulation, can be detected in patient data sets (scp). In this study we compared the results of the sca with the pressure reactivity index (PRx), an established marker for impaired autoregulation. Mean PRx values were significantly higher in time segments identified as scp compared to segments showing no selected correlations (nsc). The sca based approach predicted cerebral autoregulation failure with a sensitivity of 78.8% and a specificity of 62.6%. Autoregulation failure, as detected by the results of both analysis methods, was significantly correlated with poor outcome. Sca of brain monitoring data detects impaired autoregulation with high sensitivity and sufficient specificity. Since the sca approach allows the simultaneous detection of both major pathological conditions, disturbed autoregulation and reduced compliance, it may become a useful analysis tool for brain multimodal monitoring data.

## 1. Introduction

Neurocritical care management of patients with life-threatening diseases of the nervous system aims to avoid secondary brain injury [1]. This requires early detection of physical and biochemical events leading to increased intracranial pressure (ICP), insufficient cerebral blood flow, and brain hypoxia [2]. Since clinical signs of deterioration are confounded by therapeutic interventions such as sedation and analgesia, successful treatment interventions depend on continuous, real-time measurements of a variety of physiologic parameters [3]. However, the clinician may be confronted with more than 200 variables when evaluating a patient leading to an information overflow with a risk of misguided management decisions [4]. Consequently, in a recent consensus statement of the Neurocritical Care Society, the need for innovative data interpretation systems for early visualization of changes in the cerebral autoregulation and intracranial compliance status was defined to optimize treatment of each individual patient [5]. To generate a tool set which might facilitate detection of critical worsening of the patient, a mathematical, compartmental model of the brain focusing on slow dynamic variations was developed. This model allows simulating changes of brain multimodal monitoring data triggered by pathophysiological events such as reduced intracranial compliance and impaired autoregulation [6, 7]. In a second step, we attempted to detect the pattern of brain monitoring parameter changes predicted by the model upon simulation of impaired autoregulation in patients' data sets utilizing selected correlation analysis [8, 9]. Upon recognition of a specific data pattern, this approach directly allows drawing conclusions about the status of the patient with specific focus on intracranial compliance and cerebral autoregulation. In earlier studies, a key element of cerebral autoregulation, the pressure reactivity index (PRx), was established [10]. This index reflects the changes of cerebrovascular smooth muscle tone in response to slow spontaneous changes of arterial blood pressure (ABP). Under physiological conditions, a rise in ABP will induce cerebrovascular constriction leading to reduced cerebral blood volume and decreased ICP. Technically, the index is calculated as a short-term “moving” Pearson correlation between 10-second averages of ABP and ICP waveforms over a period of 300 seconds [10]. A positive index indicates loss of vascular pressure reactivity which can be interpreted as impaired autoregulation. This approach has been extensively validated utilizing transcranial Doppler sonography and laser Doppler flowmetry [11, 12], microdialysis [13], PET-based CBF measurements [14], and experimental animal models [15]. In addition, this index has not only been established as a strong prognostic marker [16, 17] but also demonstrated to be useful for the determination of a patient specific optimal cerebral perfusion pressure (CPPopt) [18]. The goal of the current study is to compare time segments of impaired autoregulation as determined by selected correlation analyses with the results of PRx calculations at the identical observational window. We hypothesized that if our assessment regarding impaired cerebral autoregulation based on the pattern recognition approach is correct, the PRx results should confirm this with pathologically increased index results.

## 2. Methods

### 2.1. Patient Population

The study was conducted in accordance with the ethical guidelines of the University Regensburg Institutional Review Board. Informed consent was obtained from the patient's relatives; all study results were stored and analyzed anonymously. We collected data from 29 patients (13 female, 16 male) who were treated for subarachnoid hemorrhage (*n* = 19) or traumatic brain injury (*n* = 10) at our neurosurgical ICU and who underwent brain multimodal monitoring. A detailed description of patient characteristics is provided in Table 1. The mean age was 44.8 years (range: 72.4–16.4); the median Glasgow Coma Scale GCS at the time of admission was 7 (range: 3–14). All patients were sedated and mechanically ventilated during the observation period. ICP monitoring was performed using either an external ventricular drain (EVD) or a parenchymal ICP probe (Raumedic, Helmbrechts, Germany); 10 patients (34.5%) received decompressive craniectomy due to severe brain swelling. Follow-up was completed up to October 2015 by reviewing outpatient records and contacting the patients, a family member, or the patient's primary physician. The mean follow-up time was 48.3 months; no patient was lost for follow-up. The neurological outcome was measured by the Glasgow Outcome Scale (GOS) at the last follow-up; the median score at the last follow-up was 3 (range: 1–5, Table 1).

#### 2.1.1. Selected Correlation Analysis

Selected correlation analysis (SCA) [8] represents the combination of a windowing technique with multiple frequency domain analysis (FDA) to detect correlations between two time series in a time-resolved way. In simple terms, frequency domain analysis decomposes a time series into a bunch of sinusoidal waves with fixed amplitude and frequency. Subsequently, information about relevant components of the signal or correlated components of two signals is identified. For this kind of analysis we use the so-called multitaper method (MTM), a sophisticated technique to calculate robust power spectra and coherences from short and noisy time series. To realize a time resolution, the time series are split into consecutive segments of fixed length. A pair of such segments, or windows, is used to calculate multitaper power spectra of each window and the multitaper coherence spectrum between both windows. From this spectral information, an index called selected correlation (sc) is deduced that reflects the strength of correlation between the data segments within a specific frequency band. Additionally, a differentiation between a positive correlation (scp) and a negative one (scn) is achieved by calculating the mean Hilbert phase difference of this segments.

As input data we have used synchronously recorded time series of ABP and ICP data, resampled to a sampling rate of 0.2 Hz. A correlation was called positive (scp) if the sc index was higher than a predefined limit of 0.0555556, and the mean Hilbert phase difference (mhpd) was lower than 70°. Conversely, if the sc index was below the mentioned limit and the Hilbert phase difference was between 70° and 110°, the segment was classified as uncorrelated (no selected correlation = nsc).

#### 2.1.2. Mathematical Framework

A detailed description of the mathematical methods is published in in a previous paper [19]; a brief summary is provided as follows.

Let *X*≔{*x*_1_,…, *x*_*N*_} be a time series of length *N*. A window of this time series can be represented as a function in time, defined by its starting point *k* and its length* s*:

(1) Xs,kt≔xk+t−1∈X ∣ 1≤t≤s;k+s−1≤N;s≡2u∈N.

Using such windows as input for frequency domain analysis by computing a multitaper power spectrum (mtms) of one window or the multitaper coherence spectrum (mtmc) of a pair of windows will transform the discrete time domain of the time series, indexed by* t*, into the discrete frequency domain of the spectra, indexed by *f* with range 1 ≤ *f* ≤ *s*/2. Hence we define two functions realizing this transform:

(2) Ssk,f≔mtms of Xs,kt,Csk,l,f≔mtmc of Xs,kt,Ys,lt.

MTM contains a significance test which includes a number of different significance levels. Therefore, every specific frequency *f* can be tested for significance. Based on this, we proceed to define a tuple called pointwise selected correlation (PSC) assuming a fixed significance level *C*:

(3) PSCsk,l≔psc1,…,pscs/2with: pscf≔1if Ssk,f∧Ssl,f∧Csk,l,fsignificant0otherwise.

The requirement to be significant in both spectra ensures that only frequencies are considered, which essentially contribute to the original signals. In case of the coherence spectrum, this requirement assures that a specific *f* shows a correlation between the input signals. From the above-mentioned PSC, we can calculate *n* successive pairs of isochronous windows leading to the mean pointwise selected correlation (MPSC):

(4) $$\begin{array}{c} {MPSC}^{s}\left(\;f\;\right)≔\left(\;\frac{1}{N}\;\right)*\overset{j=N}{\underset{j=1}{\sum }}{PSC}_{f}^{s}\left(\;j,j\;\right). \end{array}$$

MPSC describes the fraction of a significant occurrence in both spectra and the coherence calculation for each single frequency *f*. Utilizing MPSC it is possible to identify frequency intervals that contain the information about potential correlations within a data set. Following the identification of such a frequency interval *U* = (*m*,…, *n*), it is possible to determine periods in the data set where strong correlation with respect to *U* occurs. Subsequently, we determine the correlation strength of a distinct pair of windows with respect to *U* by calculating the sum of all elements of PSC belonging to the frequency band *U*. This sum, divided by the length of *U*, is called selected correlation (sc):

(5) scs,m,nk,l≔1n−m+1∑f=mf=nPSCfsk,lwith: 1≤m<n≤s2.

If the above-mentioned sc exceeds a predefined limit lsc, this pair of windows is classified as selected correlation. To obtain time-resolved information about the selected correlation we determine the index ${sc}^{s,m,n}\left(\tilde{t}\right)$ for isochronous windows while shifting the starting point $\tilde{t}$ along the time axis:

(6) scs,m,nt~≔1n−m+1∑f=mf=nPSCfst~,t~with: 1≤t~≤N−s+1.

In addition, we established a statistical test which calculates error rates of false positives to determine the significance of the threshold lsc.

### 2.2. Statistical Test

The statistical test for significance of lsc is based on the model prediction of isochronous correlations between ABP and ICP. In accordance with this, two segments should not be correlated if they are not isochronous. Having found a meaningful lsc we can count how often separated windows produce sc values higher than lsc. The number of wrong hits is interpreted as the error rate of sc with respect to lsc. Now we introduce the so-called mean windowed autocorrelation (mwa) to identify a sufficient offset between the input windows, so that autocorrelation effects vanish:

(7) mwas,R,m,no≔1R∑i=1i=Rscs,m,nki,ki+owith: ki random.

For sufficiently large offset *o*, the subsequent mwa values should be small and stable as autocorrelation artifacts should be excluded. Subsequently, we can calculate the error index ei^*s*,*m*,*n*^(*k*, *l*, lsc), indicating whether the selected correlation sc^*s*,*m*,*n*^(*k*, *l*) is higher than a predefined limit lsc and the error rate asc:

(8) eis,m,nk,l,lsc≔1if scs,m,nk,l>lsc0otherwise,ascs,R,m,n,olsc≔1R∑i=1i=Reis,m,nki,li,lscwith: ki random :li>ki+o.

Accordingly, a pair of isochronous data segments is called significantly correlated if the sc value of this pair is higher than the predefined limit lsc. The significance of this correlation is specified by the error rate of the given limit lsc.

### 2.3. Hilbert Phase Differences

Having identified a pair of windows exhibiting a sufficient high correlation index sc, we have to determine the phasing between the two data windows. This is done by calculating the mean Hilbert phase difference (mhpd) of the corresponding data segments, leading to values of mhpd between 0 and 180 deg. The mhpd value is based on the so-called Hilbert transform, a mathematical approach to represent a real valued function *s*(*t*) in the complex plain,

(9) sanalytict≔st+i∗s~t=At∗ei∗φtwith: s~t≔π−1P.V.∫−∞∞sτt−τdτ.

Applying this transformation to the windows *X*^*s*,*k*^(*t*), *Y*^*s*,*l*^(*t*) provides two phases *φ*_*X*_(*t*) and *φ*_*Y*_(*t*) from which we can calculate the Hilbert phase difference hpd (*t*):

(10) hpdtφXt−φYt=arctanX~s,ktYs,lt−Xs,ktY~s,ltXs,ktYs,lt−X~s,ktY~s,lt.

As we are thinking in windows here, the mean value mhpd of hpd(*t*) will serve as a measure for the phasing of two data windows:

(11) $$\begin{array}{c} {mhpd}^{s}\left(\;k,l\;\right)≔\left(\;\frac{1}{s}\;\right)\cdot \sum_{t}hpd\left(\;t\;\right). \end{array}$$

Now the selectivity of the mhpd value can easily be determined by adapting the above described error rate calculations for the sc value to mhpd by substituting the lsc criterion with appropriate criteria called lmhpd_pos_ and lmhpd_neg_. If sc > lsc and mhpd < lmhpd_pos_ the correlation between the data will be called positive (scp). If sc > lsc and mhpd > lmhpd_neg_ the correlation between the data will be called negative (scn). Conversely, if sc ≤ lsc and lmhpd_pos_ ≤ mhpd ≤ lmhpd_neg_ the segment is classified as not correlated (nsc).

### 2.4. Pressure Reactivity Index and Comparison to scp Results

A total of 550 time segments with and without selected correlation based prediction of failed autoregulation were analyzed by calculating the PRx in the identical periods. The entire observation time of all time segments was 1294.1 hours. Time segments including series with positive selected correlation (scp) between ABP and ICP indicative for perturbed autoregulation (*n* = 280) and no selected correlation (nsc; *n* = 270) were imported into ICM+ software (Cambridge Enterprise, Cambridge, UK, http://www.neurosurg.cam.ac.uk/icmplus/) [20]. The mean duration of scp segments was 3.2 hours (range: 1.6–51.6 hours); the mean nsc segment duration was 3.0 hours (range: 1.6–5.6). PRx was calculated as a short-term moving Pearson correlation coefficient of 10 s averages of ABP and ICP over 5 minutes. Utilizing a PRx value higher than 0.3 as threshold for the detection of impaired autoregulation, sensitivity, specificity, and positive (PPV) and negative predictive value (NPV) were calculated for the selected correlation analysis results. For outcome prediction, the mean PRx values of each GOS class at follow-up were compared. In addition, the percentage of time patients showed scp within the entire observation phase was correlated to GOS at follow-up by computing Spearman's rank correlation analysis.

### 2.5. Statistics

Raw data was tested for normal distribution using Kolmogorov–Smirnov test and averaged per segment prior to statistical analysis. Two- and multiple-group comparison was performed by computing a Wilcoxon rank sum test and one-way ANOVA on ranks, respectively. Association between patient's outcome and impaired autoregulation as detected by scp was analyzed by Spearman's rank correlation analysis; for prognostic impact of PRx, the mean PRx values of each GOS class were compared.

## 3. Results

**Figure 1:**
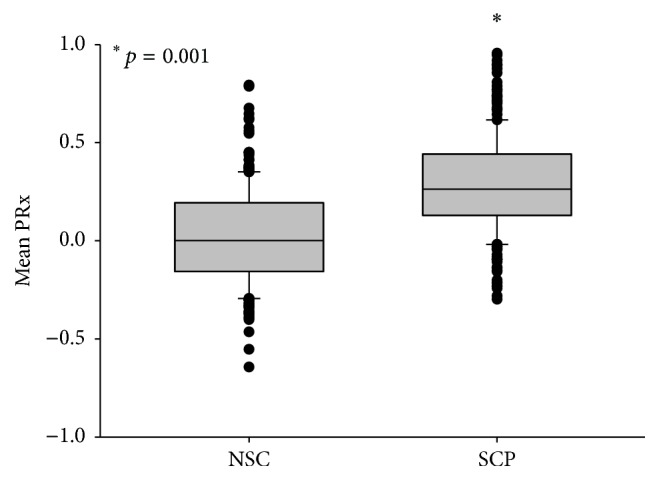
PRx values in time segments with selected correlation positive (scp) indicating impaired autoregulation are significantly higher compared to no selected correlation (nsc) (mean: 0.026 versus 0.286; *p* = 0.001).

**Figure 2:**
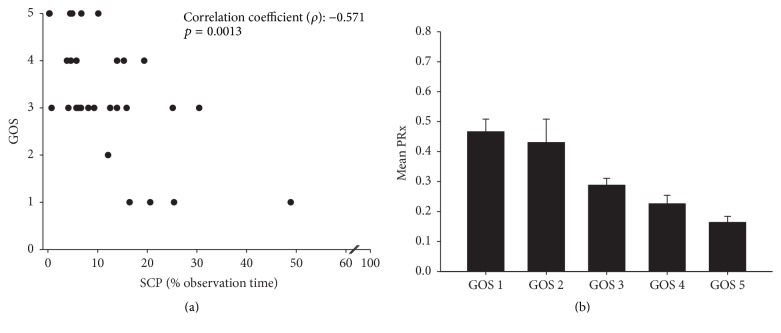
Outcome studies. (a) The percentage of time during the observational period in which patients showed scp, indicating a disturbed autoregulation, strongly correlates with outcome measured by GOS at the last follow-up (*p* = 0.0013; Spearman's rank correlation analysis). (b) Correspondingly, high PRx values implicating deteriorated cerebrovascular pressure reactivity were associated with poor outcome (ANOVA on ranks, *p* < 0.001).

PRx values were significantly higher in the time segments with scp compared to nsc (mean: 0.286 versus 0.026; *p* = 0.001; Figure 1). Utilizing a PRx threshold value of 0.3, selected correlation analysis resulting in scp (mean Hilbert phase < 70°) predicted impairment of autoregulation with a sensitivity of 78.8% and a specificity of 62.6%. Correspondingly, the PPV of scp was 0.49; the NPV was 0.87. Mean ICP values of time segments with scp were significantly higher compared to nsc segments (14.4 versus 11.6 mmHG; *p* < 0.001). The percentage of time during the observational period in which patients showed scp, indicating a disturbed autoregulation, correlated highly significant with patient's outcome as measured by the Glasgow Outcome Scale (correlation coefficient (*ρ*) = −0.571; *p* = 0.0013; Figure 2(a)). The mean PRx values were significantly higher in patients with poor outcome and profoundly lower in patients with favorable outcome (*p* < 0.001; Figure 2(b)).

## 4. Discussion

Neuroprotection after catastrophic CNS events as a causal treatment has been the primary goal of neuroscientific research in the field; however so far clinical trials have not yielded any significant success in patients with either SAH or TBI [21, 22]. In contrast, the implementation of neurointensive care management utilizing brain multimodal monitoring in order to provide an optimal environment to prevent secondary injury has significantly improved the prognosis of patients inflicted with critical neurological disease [23–26]. Since focusing on one single parameter such as ICP may not be sufficient [27], treatment guidance requires the interpretation of high resolution data sets derived from a multitude of different monitoring devices in order to unmask complex pathophysiological events [3]. In order to develop a bioinformatics tool set for the integrative real-time analysis of brain monitoring data, we have established a mathematical model, which allows the simulation of pathological conditions such as impaired autoregulation or exhausted intracranial compliance [6]. Identical changes of monitoring data resulting from this model were detected in patients utilizing the selected correlation analysis, allowing conclusions regarding the intracranial compliance [9] and function of autoregulation [19]. The comparison of our results with the pressure reactivity index (PRx) established by Czosnyka and coworkers [10] revealed significantly higher PRx values in the time segments with impaired autoregulation as detected by selected correlation analysis. Using a PRx threshold of 0.3, our approach detected changes in autoregulation with a sensitivity of 78.8%. In addition, both approaches resulted in a strong correlation between detected changes of autoregulation and patients outcome. As a limitation of our study, the size of our cohort is relatively small and does not allow extensive statistical analysis of additional outcome parameters. However, we could demonstrate that both approaches, scp and PRx, are related to outcome in a similar fashion, indicating that impaired autoregulation over a prolonged period of time is associated with poorer outcome. It has been demonstrated that changes of intracranial compliance, for example, following decompressive craniectomy, may have significant impact on the PRx including the determination of CPPopt [28]. While Timofeev al. [29] found increased PRx values following craniectomy, two other studies found significantly reduced values [30, 31]. The major reason for this effect is that, after craniectomy, the intracranial compliance is artificially increased, which results in a flattened cerebrospinal pressure-volume curve which impedes the transmission of blood volume changes into ICP. This in turn may potentially reduce the sensitivity of the PRx towards an impaired autoregulation. It is therefore of paramount importance to implement the aspect of intracranial compliance into the analysis of autoregulation function. Since the sca approach allows the simultaneous detection of both major pathological conditions, reduced intracranial compliance [9] and autoregulation failure, it may offer an optimized platform for a computerized bedside monitoring of patients with critical neurological diseases.

In conclusion, selected correlation analysis of brain monitoring data detects impaired autoregulation with high sensitivity and sufficient specificity when using the established index PRx as comparator.

## Figures and Tables

**Table 1:** Characteristics of patients, receiving treatment for either SAH (subarachnoid hemorrhage) or TBI (traumatic brain injury) who were included in the study. The initial levels of consciousness at admission according to the Glasgow Coma Scale is depicted in column GCS; patients receiving decompressive craniectomy due to brain swelling are indicated by “y”; the next column describes the brain monitoring time for each patient in hours; outcome at last follow-up is noted in column GOS. The mean ICP and PRx columns describe the mean ICP and PRx values averaged over the entire observation time per patient.

Gender	Age	Diagnosis	GCS	Decompressive craniectomy	Monitoring time (hours)	GOS (last follow-up)	Mean ICP (mmHG)	Mean PRx
M	44.1	SAH	3	n	25.9	5	7.6	0.177
M	60.2	TBI	10	n	73.6	3	13.2	0.263
M	72.4	TBI	14	y	115.9	3	8.4	0.253
M	21.9	TBI	3	y	126.9	5	11.5	−0.078
M	51.6	SAH	8	n	136.7	1	20.8	0.412
F	51.6	SAH	9	n	162.6	3	9.7	0.255
M	65.4	TBI	6	y	184.3	3	9.7	0.172
F	26.2	TBI	7	y	187.2	5	14.8	−0.030
M	53.0	SAH	3	n	193.3	1	19.6	0.388
M	43.4	SAH	3	y	212.7	4	9.3	0.229
F	50.4	SAH	14	n	217.7	5	11.0	0.159
F	38.4	SAH	12	n	248.3	3	9.0	0.047
F	49.0	SAH	6	n	256.3	4	11.2	0.281
M	35.4	TBI	7	n	262.3	4	11.4	0.265
F	43.4	SAH	11	n	262.6	1	15.6	0.341
F	58.4	SAH	3	n	280.9	3	12.8	−0.008
M	18.5	TBI	3	y	289.8	3	11.4	−0.177
F	32.3	SAH	3	n	294.8	5	15.7	0.151
M	16.4	TBI	3	n	321.9	2	9.9	0.161
F	65.8	SAH	14	y	336.4	1	25.1	0.177
F	42.4	SAH	14	n	351.9	3	9.5	0.118
M	36.5	TBI	8	y	379.2	5	12.9	0.138
F	26.5	SAH	3	n	383.5	3	16.4	0.242
M	59.3	SAH	6	n	386.6	4	10.0	0.232
M	36.3	SAH	12	n	404.2	3	12.5	0.133
M	33.6	TBI	3	n	404.5	4	11.4	−0.152
F	62.8	SAH	11	y	453.9	3	10.7	−0.003
M	51.0	SAH	7	n	484.5	3	15.3	0.235
F	52.1	SAH	3	y	600.2	4	8.6	0.034
